# To serve or to leave: a question faced by public sector healthcare providers in Pakistan

**DOI:** 10.1186/s12961-015-0045-4

**Published:** 2015-11-25

**Authors:** Ali Mohammad Mir, Muhammad Saleem Shaikh, Gul Rashida, Neha Mankani

**Affiliations:** Population Council, Islamabad, Pakistan; Aahung, Karachi, Pakistan

**Keywords:** Female, Health, Motivation, Pakistan, Providers, Rural

## Abstract

**Background:**

The availability of properly trained and motivated providers is a prerequisite for provision of easily accessible healthcare. Pakistan has been listed by the World Health Organization in its World Health Report 2006 as one of 57 countries with a critical health workforce deficiency. This study examines the factors associated with the willingness of public sector healthcare providers to leave government service and recommends measures that can be adopted to attract and retain staff in the country’s public healthcare system.

**Methods:**

A stratified, random sampling methodology was adopted to recruit a nationally representative sample of 1,296 public sector healthcare providers, including paramedics, medical doctors, and specialists. A semi-structured questionnaire was used to interview these providers. Logistic regressions measured the association with determinants of their willingness to leave the public health sector for better prospects elsewhere.

**Results:**

A third of all healthcare providers who were interviewed were of the view that, provided the opportunity, they would leave government service. The odds of willingness to leave service were highest among providers from the region of Azad Jammu and Kashmir (adjusted odds ratio [AOR] = 4.33; 95% CI, 2.49–7.54) followed by the province of Balochistan (AOR = 4.21; 95% CI, 2.41–7.33), and the region of Gilgit Baltistan (AOR = 3.34; 95% CI, 1.67–6.67). Providers who expressed dissatisfaction in the manner their performance was evaluated and those who were dissatisfied with the current salary, each had higher odds of considering leaving government service (AOR = 1.67; 95% CI, 1.18–2.40 and AOR = 2.03; 95% CI, 1.47–2.81, respectively). Providers who reported experiencing interference in their work by influential politicians of the area were more inclined to leave (AOR = 1.44; 95% CI, 1.05–1.98).

**Conclusion:**

This study clearly highlights the need to implement more focused strategies in the public healthcare system in Pakistan in order to build sufficient staff motivation and prevent providers from leaving government service. In order to improve coverage of healthcare services in Pakistan, the government will have to introduce more focused interventions to attract and retain healthcare providers, especially in remote and rural areas of the country.

## Background

Pakistan is among the few countries in the region that have failed to achieve their Millennium Development Goal targets, with the country lagging behind many of its neighbours in terms of improving its reproductive health indicators. The lack of human resources is one of the major constraints compromising the effectiveness of its public healthcare system [1,2].

Staff attrition is caused by a variety of factors related to job satisfaction and employee motivation, including the level of workers’ autonomy, delegation of responsibility, and authority afforded through regular promotion, as well as the relative status of the worker within and outside the organization [3]. Attrition or staff turnover not only compromises the quality of care [4], but also has cost implications for the health system as a whole due to the expenses associated with the recruitment and training of new staff [5]. Preventing attrition can therefore be a key strategy in enhancing the effectiveness and efficiency of the healthcare system.

Studies conducted in low- and middle-income countries have emphasized the difficulty in recruiting staff to the public sector (and to rural areas) and in their subsequent retention [6]. The combination of a public sector job in a rural setting is unattractive to many, especially when provided the opportunity to work in cities, of greater income through private clinics, and of a better standard of living [6]. The ability to generate additional income on top of a government salary is an important factor underlying the preference for urban workplaces: public health sector salaries are not sufficient and there is more of a potential market for private practices in urban areas. Further, working conditions in rural public health facilities tend to be much worse than in urban facilities [7].

Farooq et al. [8] found that doctors from Pakistan with a relatively high social and economic status were unwilling to work in public sector rural facilities because they felt it would have a negative impact on their professional and family lives. These workers require incentives other than residential facilities in order to serve in remote areas, including transportation assistance (in cases where there are no residences in the health facility) and the assurance of their personal and family safety [9-12]. In this global (and regional) context of increasing inequities, health service policymakers are trying to increase staff retention in inaccessible and socioeconomically poor areas. In order to prevent attrition among staff, the government of Pakistan has introduced a number of changes in recent years, including incentives such as better salaries and additional rural and/or cold area allowances.

The research study described herein is part of a larger study performed to produce insightful and policy-relevant evidence on the range of factors that constrain mid-level providers from or motivate them to serve in key positions in public health facilities in rural Pakistan [13]. This research tackled the confluence of job-related, institutional, demographic, social, and economic issues that health workers face in rural Pakistan. In the present paper, we have examined the willingness of public sector providers to shift to the private sector when presented with the opportunity and the factors associated with their willingness to do so. Further, we have examined the willingness to leave service as an intervening variable between job satisfaction and actual turnover [14].

## Methodology

A nationally representative, cross-sectional survey of healthcare providers was carried out between September 20 and November 15, 2012, covering all administrative units of Pakistan. The survey assessed the issues of retention and motivation among public sector healthcare providers in Pakistan.

### Sampling

A stratified, random sampling strategy was employed to identify the study districts in each administrative unit. The administrative units of Pakistan consist of four provinces, Punjab, Sindh, Baluchistan, and Khyber Pakhtunkhwa, and three non-provincial units, Azad Jammu and Kashmir (AJK), Gilgit-Baltistan (GB), and a group of Federally Administered Tribal Areas (FATA). We divided the country into seven strata, including North Punjab, South Punjab, Sindh, Khyber Pakhtunkhwa, Balochistan, AJK, and GB and FATA. Punjab was assigned two strata due to the vast difference in health outcomes in the northern and southern parts of the province. GB and FATA were collapsed into one stratum due to the paucity of healthcare providers in these areas.

Districts in each of the strata were arranged according to socioeconomic index ranking and level of skilled birth attendance. Four districts were then randomly selected from each stratum, including one each from the upper and middle socioeconomic bands and two districts from the lower rungs of district ranking to over-represent the less served or underserved areas in each stratum. Thus, a sample of 28 districts was identified.

Within each district, health facilities were randomly selected, with the proportion of different types of facilities in the sample representing the actual proportion of such facilities in the district. The different types of health facilities included district headquarter hospitals, which are tertiary facilities; tehsil headquarter hospitals, secondary level facilities; and rural health centres, basic health units, and mother and child healthcare centres, which provide primary healthcare. In each district, there is usually one district headquarter hospital, two to three tehsil headquarter hospitals, five to 10 rural health centres, and about 50 basic health units. Accordingly, in each district, we selected all district headquarter hospitals, two tehsil headquarter hospitals (i.e. nearly 50% of such facilities), three rural health centres, 10 basic health units (i.e. nearly 20% of all such facilities), all mother and child healthcare centres, and the main tertiary care hospital catering to gynaecology/obstetric patients. Overall, 533 facilities were visited.

Within the facilities, interviews were conducted with the following pre-identified categories of providers: (1) specialists, including gynaecologists and paediatricians (posted mainly at the district headquarter hospitals, tehsil headquarter hospitals, and some rural health centres); (2) male medical officers, i.e. male doctors with postgraduate training; (3) women medical officers, i.e. female doctors with postgraduate training; (4) Lady Health Visitors (LHVs) and nurses; and (5) technicians and dispensers.

At each of the selected facilities, interviews were conducted with all specialists and one provider from each of the other four categories, selected through random sampling. Overall, interviews were conducted with 1,296 healthcare providers, including 22 medical superintendents, 87 specialists (gynaecologists and paediatricians), 242 medical officers, 130 women medical officers, 503 LHVs and nurses, and 312 technicians and dispensers. In addition, 69 hospital managers and administrators were also interviewed. In case respondents were not available on the first visit, three subsequent visits were conducted by the study team to interview them.

### Questionnaire

A semi-structured questionnaire was used to conduct interviews. Previously validated questions [15,16] were adapted to the Pakistani context. The questions were related to various aspects of job satisfaction and probed, for example, the reasons for career selection, motivation, organizational commitment, issues of safety and security, and work environment perceptions. The questionnaire also included a section for measuring respondents’ job satisfaction using the Job Descriptive Index tool developed at Bowling Green State University, USA in 1969 [17], which has since been used extensively worldwide [18].

The questionnaire was first translated into Urdu and retranslated into English to ensure consistency; the final version was in both languages. The questionnaire was pilot-tested in two non-sampled districts, Rawalpindi and Jhelum. In total, 14 structured questionnaires were completed by healthcare providers of different cadres. The weaknesses of the questionnaire, mainly related to the language and sequencing of questions, were discussed, and the questionnaire was accordingly refined by the Population Council’s research team.

The interviewers who administered the questionnaire all held master’s degrees in the social sciences and were extensively trained to conduct the interviews so as to minimize measurement error and social desirability bias.

### Variables and hypotheses

The primary outcome of the current study – the willingness to leave government service – was assessed by asking study participants whether they would consider shifting to the private sector if provided the opportunity. According to Herzberg, as quoted by Franco et al. [3], if employees feel that their basic needs are not being met, such as when salary is inadequate, work conditions are not safe, or organizational policies are not being properly followed, they may consider leaving their present job. In this study, we considered basic demographic features (e.g. age and gender), professional cadre, satisfaction with current level of salary, promotion status and policies, and work-related interference by local politicians as the factors associated with willingness to leave. Promotion is made based on the Annual Confidential Report and we hypothesised that dissatisfaction with the report may influence a provider’s willingness to leave; one promotion was equivalent to 5 years of service. Political interference was defined as undue interference in provider’s routine work by influential politicians of the area.

### Data collection and analysis

Data was collected using the CSPro 3 data entry programme and analysed using the Statistical Package for the Social Sciences version 14. The data manager conducted quality checks on questionnaire completeness, inter-record checks, and mistakes in data files, and suggested corrections in consultation with the principal investigator.

Univariate analysis was first performed to describe the sample. Significant relationships at the bivariate level were further analysed using logistic regression at the multivariate level to examine the relationship between key independent variables with willingness to leave.

### Ethical considerations

Ethical approval was obtained from the Institutional Review Board of the Population Council’s headquarters in New York and by the National Bioethics Committee of Pakistan. Written informed consent was obtained from all study participants after a detailed description of the study. Prior appointment was obtained to set up each interview at the most convenient time for the respondent. Usually, the interviews took place when the providers were free from their routine duties. The interviews were held mainly within the workplace and privacy was ensured by the interviewers.

The interviewers described the scope and purpose of the questionnaire and its approximate length, and stressed that participation was voluntary. The respondents were assured about the confidentiality of their responses and that the information being provided would not be attributed to them by name.

## Results

The response rate was 94%, while the refusal rate was less than 6%; refusals were random and did not represent any specific category of providers. The number of interviews that took place depended on staff availability at the facility at the time of the visit.

### Sociodemographic characteristics of respondents

Table 1 summarizes the basic demographic characteristics of the respondents. Of the providers interviewed, 76% were regular staff, while the rest were employed on short-term contracts. Slightly more than half of the respondents (52%) were female.

**Table 1:** **Sociodemographic characteristics of respondents**

	**Description of the sample**	**n**	**%**
Gender			
	Male	624	48
	Female	673	52
Age, years			
	Less than 25	112	9
	26–35	453	35
	36–45	427	33
	46 and above	304	23
Marital status			
	Unmarried	226	17
	Married	1047	81
	Divorced	7	1
	Widowed	14	1
Healthcare provider type			
	Medical superintendants	22	2
	Specialists	87	7
	Medical officers	242	19
	Women medical officers	130	10
	Lady health workers	403	31
	Nurses	100	8
	Technicians/dispensers	312	24
Residential status			
	Live within the premises of the facility	255	20
	Live within the city where they work	743	57
	Live in another city	295	23
Region			
	Punjab North	231	18
	Punjab South	201	15
	Sindh	202	16
	Khyber Pakhtunkhwa	179	14
	Federally Administered Tribal Areas	87	7
	Gilgit-Baltistan	90	7
	Balochistan	140	11
	Azad Jammu and Kashmir	172	13
Contractual status			
	Contract staff	313	24
	Regular staff	971	76
Salary satisfaction			
	Satisfied with salary	507	39
	Dissatisfied with salary	785	61
Promotion status			
	Never promoted	790	61
	1 promotion	420	32
	2 promotions	69	5
	3 promotions	23	2
Number of years’ experience			
	Less than 5	321	25
	6–10	209	16
	11–15	199	15
	16–20	196	15
	21 and above	363	28
Political interference			
	No	827	64
	Yes	465	36
Satisfaction with the Annual Confidential Performance Report			
	No	274	27
	Yes	743	73

The mean age of the respondents was 38 years and four-fifths were married. LHVs comprised the largest category of respondents, followed by dispensers/technicians. Only a fifth of the providers resided within the premises of the health facility where they were posted.

### Willingness to leave government service

#### Bivariate analysis

Nearly a third of all providers (n = 425) reported that they would consider leaving government service (data not shown). The most cited reasons were better salary prospects (87%) in the private sector, followed by easier commuting to work (30%), better work environment (27%), and a lack of political interference in the performance of duties (21%) in the private sector.

At the bivariate level, the proportion of male providers (39%) who would consider leaving government service was greater than that of female providers (27%). The proportion of staff who reported that they would consider moving to the private sector was highest among medical officers (44%), followed by specialists (36%), women medical officers (36%), and LHVs and nurses (24%). Among the respondents who reported that they would leave their present job if an opportunity was made available, the highest proportion (71%) was of workers who were dissatisfied with their salary.

Of providers who would leave, two-thirds had never received a promotion, 5% had received two promotions, and only 1% had received three promotions. The association between promotion status and willingness to leave for the private sector was statistically significant (*P* <0.01). Among providers who had served for 0–5 years, there was a higher proportion expressing a willingness to leave (28.2%) compared to those who had worked for more than 10 years (17.2%; *P* <0.05). This may be related to organizational loyalty and greater organizational commitment.

Dissatisfaction with the work assessment process also contributed to willingness to leave; providers who were satisfied with the Annual Confidential Report were significantly less likely to leave (38%; *P* <0.01). Providers who reported experiencing political interference (56%) were more likely to be willing to leave than those who did not (44%; *P* <0.05).

#### Multivariate analysis

As seen in Table 2, compared to the reference group of male medical officers, nurses and LHVs were less likely to leave (adjusted odds ratio (AOR) = 0.26; 95% confidence interval (CI), 0.114–0.603 and AOR = 0.42; 95% CI, 0.269–1.660, respectively). Salary dissatisfaction also contributed to willingness to leave. Providers who were dissatisfied with their salary were twice as likely to leave (AOR = 2.03; 95% CI, 1.47–2.81; *P* <0.01) than those who were satisfied. However, promotion and work experience had no significant relationship with willingness to leave. Dissatisfaction with the work assessment process also contributed to willingness to leave; providers who were satisfied with the Annual Confidential Report were significantly less likely to do so (AOR = 1.67; 95% CI, 1.18–2.40; *P* <0.01). Providers who reported experiencing political interference had higher odds of leaving than those who did not (AOR = 2.03; 95% CI, 1.05–1.98; *P* <0.05). Providers from AJK, Balochistan, and GB had significantly higher odds (*P* <0.001) of expressing their willingness to shift to the private sector (AJK: AOR = 4.33; 95% CI, 2.49–7.54; *P* <0.01; Balochistan: AOR = 4.21; 95% CI, 2.41–7.33; *P* <0.01; GB: AOR = 3.34; 95% CI, 1.67–6.67; *P* <0.01).

**Table 2:** **Multivariate logistic regression models of predictors of willingness to leave government service if an opportunity arose**

**Variable**	**Adjusted odds ratio (95 % CI)**
**Cadre**	
Male medical officers	1.00
Lady health Visitors	0.42 (0.269–0.660) ***
Specialists	0.66 (0.360–1.215)
Nurses	0.26 (0.114–0.603) ***
Women medical officers	0.92 (0.505–1.693)
Technicians/dispensers	0.77 (0.494–1.20)
Medical superintendents	0.64 (0.222–1.823)
**Promotion**	
Yes	1.00
No	1.03 (0.74–1.42)
**Experience, years**	
Less than 5	1.00
5–10	1.20 (0.71–2.02)
11–15	0.90 (0.52–1.56)
16–20	1.07 (0.62–1.83)
21 and above	0.75 (0.453–1.24)
**Region**	
Punjab North	1
Punjab South	1.79 (1.04–3.09) **
Sindh	2.16 (1.23–3.82) **
Khyber Pakhtunkhwa	2.84 (1.59–5.05) ***
Federally Administered Tribal Areas	2.77 (1.35–5.71) **
Gilgit Baltistan	3.34 (1.67–6.67) ***
Azad Jammu and Kashmir	4.33 (2.49–7.54) ***
Balochistan	4.21 (2.41–7.33) ***
**Satisfaction with Annual Confidential Report**	
Yes	1.00
No	1.67 (1.18–2.40) ***
**Political interference**	
No	1.00
Yes	1.44 (1.05–1.98) **
**Satisfaction with salary**	
Yes	1.00
No	2.03 (1.47–2.81) ***

**P <0.05, ***P <0.01.

## Discussion

Healthcare staff attrition is an area of major concern since it is inversely correlated to job satisfaction [19-22]. A third of the providers interviewed in this cross-sectional survey said that they would consider leaving government service if provided the opportunity. These findings are important for Pakistan as the country is already facing a persistent shortage of health workers to serve in rural areas. The multivariate logistic regression model analysis shows that the odds of considering leaving government service were highest among providers in AJK, followed by Balochistan and GB. Topographically, these areas have harsh working conditions due to severe winters and a mountainous terrain. Incentivizing providers to work in these areas would require establishing proper residential facilities at the place of their posting and enhancing their level of comfort.

Salary dissatisfaction was also an important factor associated with health workers’ willingness to leave. Earlier studies have found that low salary and inadequate allowances lead to staff dissatisfaction, attrition, absenteeism, and poor quality of care [23-26]. Previous studies conducted in Faisalabad and Karachi in Pakistan have also found that the majority of providers believe that they are paid less than their skills and qualifications merit or that the facilities and benefits they receive are inadequate [27,28]; further, pay and benefits were also often at the top of the list of reasons for dissatisfaction among health professionals. While a substantial increase in salary may not be feasible for the government when it is facing an economic crisis, there should be a proposal to at least make the pay package of specialists more lucrative, especially for those who opt to work at rural health centres.

While a career structure for health workers does exist, according to the majority of respondents, it is not being adhered to, mainly due to political interference and a poor mechanism to measure and evaluate performance. Political interference here implies pressure exerted by ruling politicians to grant undue favours to candidates of their choice. The main problems associated with the current evaluation process of public sector providers is that the pro-forma used for assessing performance is uniform for all government sector employees and therefore does not capture the distinct nature of work or accomplishments of healthcare staff.

The results presented herein are a component of a larger national study [13] assessing staff motivation. We acknowledge that a major limitation of this study is that it does not capture the perception of providers who have already left government service. Instead, it focuses on the perceptions of providers who remain within the system and who may therefore have higher levels of motivation than those who have left. Secondly, it is based on cross-sectional rather than prospective measurement of attrition; the latter approach would have helped in linking willingness to leave with actual departure from government service [29,30]. To overcome the possibilities of social desirability bias influencing the study findings we ensured that the interviewers took great care in assuring the respondents about the confidentiality of their responses.

### Recommendations

Based on the study findings, the following recommendations are proposed, in consultation with provincial and district managers, to prevent staff attrition:

- All provinces must put into effect a Human Resource Development Cell and ensure implementation of a “Human Resources for Health Management” policy; strategies should be developed to implement specific interventions that respond to the issues identified in this study. There is also a need for certain administrative reforms. The existing performance evaluation system needs to be revised by identifying new, job-based indicators for performance evaluation and incorporating these indicators in the forms used to compile the Annual Confidential Report. A system of periodic examination of providers should be introduced, similar to the system in the Armed Forces of Pakistan, to objectively measure staff competence in relation to the specific requirements of their current position and career structure. According to study respondents, greater adherence to organizational policies should also be stressed to minimize political interference.
- A comprehensive rural services package needs to be developed that clearly incentivizes and makes working in the rural areas more attractive, for both male and female providers. This should include monetary and non-monetary rewards, with a competitive salary package. Provincial managers have suggested dividing districts into zones based on hardship level – defined in terms of availability of schools, security situation, and road access – and offering higher financial incentives for providers posted in the tougher zones. The incentives should include increased allowances to compensate for working conditions and a travel allowance, especially for providers who have not been provided residence within the facility premises, all of which should be adjusted for inflation. Non-monetary rewards to enhance motivation can be introduced such as recognition and rewarding good performance, award of letters, certificates, or shields of appreciation to staff who show outstanding performance against key performance indicators.
- Posting in rural areas should be on a tenure basis and must be made mandatory by the Pakistan Medical and Dental Council as a requirement for registration. To increase access to specialized care in rural areas, the College of Physicians and Surgeons of Pakistan should make serving in these areas a requirement for obtaining postgraduate fellowship qualifications.
- To increase the availability of female providers, especially Women Medical Officers and female specialists in rural areas, a special tenure system should be introduced and postings made on a rotation basis. Female paramedical staff should, wherever possible, be from the same area where they are posted. Residences for female providers must be provided to ensure their safety and security as well as to enable them to be available to cater to emergency cases during late hours.

## Conclusion

In order to improve coverage of healthcare services in Pakistan, the government needs to introduce health system reforms to improve retention of healthcare providers and to help them work optimally in remote communities.

## Abbreviations

AJK: Azad Jammu and Kashmir
AOR: Adjusted odds ratios
CI: Confidence intervals
FATA: Federally Administered Tribal Areas
GB: Gilgit-Baltistan
LHVs: Lady health visitors
